# Acetaminophen Hepatotoxicity: Not as Simple as One Might Think! Introductory Comments on the Special Issue—*Recent Advances in Acetaminophen Hepatotoxicity*

**DOI:** 10.3390/livers2030008

**Published:** 2022-07-01

**Authors:** Hartmut Jaeschke

**Affiliations:** Department of Pharmacology, Toxicology & Therapeutics, University of Kansas Medical Center, Kansas City, KS 66160, USA

Acetaminophen (N-acetyl-para-aminophenol (APAP)) is one of the most-studied drugs worldwide. APAP causes liver toxicity after an overdose, with thousands of papers published on various aspects of the mechanisms of cell death and organ injury, as well as regeneration and recovery. It is also a highly popular experimental model to test the efficacy of various potential drugs and chemicals to treat or prevent acute liver injury and promote regeneration. The popularity of the APAP overdose model is derived from two main aspects: the clinical relevance of the model and the perceived simplicity of the experimental design.

Regarding the clinical relevance, APAP is present in hundreds of prescriptions and over-the-counter medicines, which are consumed daily by tens of millions of patients worldwide. Although considered safe at therapeutic doses, an overdose of APAP dose-dependently causes liver injury, which can progress to acute liver failure (ALF) and even death in patients [1,2]. In fact, APAP toxicity is the most frequent cause of ALF in the US, the UK and many other western countries [3,4]. Mitchell and coworkers [5–7] discovered that the sensitivity of mice to APAP toxicity is comparable to that of humans and defined the early steps of toxicity in the murine model. Importantly, an APAP overdose in the mouse accurately reproduces most of the mechanistic aspects of cell death and liver injury observed in patients [8] and human hepatocytes [9], with the only exception being the more delayed pathophysiology observed in humans compared to mice. Thus, the mechanistic data and therapeutic intervention strategies obtained in the mouse model translate very well to the human pathophysiology [10,11]. The only clinically approved antidote against APAP toxicity, N-acetylcysteine, was developed based on the early mechanistic insight generated by Mitchell and coworkers in the mouse model [12,13]. In addition, the most-promising new antidote under clinical development, fomepizole (4-methylpyrazole), is being advanced due to preclinical studies in the mouse model that demonstrated that the compound is an effective inhibitor of cytochrome P450 2E1 (Cyp2E1) and of c-jun N-terminal kinase (JNK) [14,15]; one aspect of this mechanism (Cyp2E1 inhibition) was confirmed in a human volunteer trial [16]. Based on this experience, APAP overdose in the mouse is the preferred experimental model to study clinically relevant mechanisms of acute drug hepatotoxicity and regeneration and evaluate potential therapeutic targets.

The second aspect that contributes to the popularity of APAP toxicity is the perceived simplicity of the model. Fed or overnight fasted mice from most mouse strains develop severe liver injury when intraperitoneally injected with a dose of 300–600 mg/kg APAP [17]. Thus, it seems simple enough to sacrifice the animals 24 h after APAP administration and measure as many parameters related to injury, modes of cell death, inflammation, oxidant stress, etc., as possible. However, this simplistic experimental design provides a substantial problem for the interpretation of the results. APAP toxicity is a complex, time-dependent process involving many different, interrelated mechanistic aspects, including drug metabolism, with the formation of a reactive metabolite, GSH depletion and protein adducts formation, an initial oxidant stress that activates a mitogen-activated protein kinase cascade leading to JNK phosphorylation, phospho-JNK translocation to mitochondria with amplification of the oxidant stress and peroxynitrite formation, and iron-dependent nitrotyrosine protein adduct formation in mitochondria, eventually leading to the mitochondrial permeability transition pore opening and collapse of the mitochondrial membrane potential [18,19]. The mitochondrial dysfunction then leads to the release of endonucleases, which cause DNA fragmentation. These are the key events leading to necrotic cell death [20,21]. However, there are many different mechanisms that can affect these central pathways of cell death, including Nrf2 activation with an impact on drug metabolism and defense mechanisms [22], autophagy and mitophagy to limit the propagation of mitochondrial damage [23], and mitochondrial biogenesis to replace damaged mitochondria, limit cell death and facilitate regeneration [24,25], and an extensive sterile inflammatory response to promote recovery. However, they may also risk aggravating the injury process under certain conditions [26,27]. In addition to these major adaptive responses to the stress of injury, there are additional aspects to consider, such as the gut microbiome, dietary effects, and genetic background, all of which could influence the pathophysiology through modulation of any of the above-mentioned effects, and thus ultimately influence cell necrosis. Although the zonation of hepatocytes has been known for many years [28], the more recent application of single-cell RNA-sequencing now allows for the response of individual hepatocytes and non-parenchymal cells to hepatotoxins such as APAP to be studied, and opens up a new dimension in the investigation of APAP hepatotoxicity [29,30]. Despite this wealth of information, there are still many open questions that need to be investigated and novel interactions that can be discovered. However, avoiding pitfalls in experimental design and mistakes in data interpretation is critical to relevant progress in this field [31].

Therefore, the objective of this Special Issue on “Recent Advances in Acetaminophen Hepatotoxicity”(https://www.mdpi.com/journal/livers/special_issues/acetaminophen_hepatotoxicity (accessed on 19 June 2022)) is to publish state-of-the-art reviews summarizing the newest developments by leading experts and attract additional reviews and original manuscripts that can further define the field, advance our understanding of the pathophysiology, and identify novel therapeutic targets.
